# Game Addiction Scale Assessment Through a Nationally Representative Sample of Young Adult Men: Item Response Theory Graded–Response Modeling

**DOI:** 10.2196/10058

**Published:** 2018-08-27

**Authors:** Yasser Khazaal, Kyrre Breivik, Joel Billieux, Daniele Zullino, Gabriel Thorens, Sophia Achab, Gerhard Gmel, Anne Chatton

**Affiliations:** ^1^ Geneva University Geneve Switzerland; ^2^ Regional Centre for Child and Youth Mental Health and Child Welfare, Uni Research Health Bergen Norway; ^3^ Addictive and Compulsive Behaviours Lab Institute for Health and Behaviour University of Luxembourg Esch-sur-Alzette Luxembourg; ^4^ Department of Mental Health and Psychiatry Geneva University Hospitals Geneva Switzerland; ^5^ Alcohol Treatment Center, Lausanne University Hospital Lausanne Switzerland

**Keywords:** internet addiction, internet gaming disorder, internet gaming, item response theory, game addiction scale

## Abstract

**Background:**

The 7-item Game Addiction Scale (GAS) has been validated under standard confirmatory factor analysis and exhibits good psychometric properties. Whether this scale satisfies the necessary conditions for consideration by item response theory (IRT) modeling remains unknown. However, the Diagnostic and Statistical Manual of Mental Disorders, 5th Edition (DSM-5) recently proposed criteria, in its section 3, to define internet gaming disorder (IGD) to promote research on this possible condition.

**Objective:**

The objective of our study was to (1) analyze GAS in the context of IRT (graded-response) modeling; (2) investigate differential item functioning (DIF), a feature of IRT modeling, in 2 subsamples; and (3) contribute to the ongoing (IGD) debate related to the validity of the DSM-5 criteria using GAS items as a proxy.

**Methods:**

We assessed 2 large representative samples of Swiss men (3320 French-speaking and 2670 German-speaking) with GAS.

**Results:**

All items comprised high discrimination parameters. GAS items such as relapse, conflict, withdrawal, and problems (loss of interests) were endorsed more frequently in more severe IGD stages, whereas items related to tolerance, salience (preoccupation), and mood modification (escape) were endorsed more widely among participants (including in less severe IGD stages). Several DIF effects were found but were classified as negligible.

**Conclusions:**

The results of the analyses partly support the relevance of using IRT to further establish the psychometric properties of the GAS items. This study contributes to testing the validity of the IGD criteria, although cautious generalization of our findings is required with GAS being only a proxy of the IGD criteria.

## Introduction

In recent years, growing concerns have been expressed concerning public health issues related to excessive internet use [1] and online gaming [2,3], leading to numerous studies and debate about the possible addictive characteristics of some behaviors associated with the excessive use of internet games [4-7]. Thus, it is crucial to better understand and screen for potential disorders such as internet gaming addiction.

Many tools have been developed to this end, including the Game Addiction Scale (GAS) by Lemmens et al [8]. GAS was created to measure the following 7 criteria: *salience*, *tolerance*, *mood modification*, *relapse, withdrawal*, *conflict*, and *problems*. Validation of GAS in 2 samples of Dutch adolescent gamers showed good psychometric properties. GAS was, subsequently, cross culturally validated with 2 independent samples from two linguistic regions in Switzerland [9]. Standard confirmatory factor analysis (CFA) results revealed that the scale behaves similarly in both regions except for one item (withdrawal). This item showed a lack of invariance.

Standard CFA and item response theory (IRT) are two popular methods for establishing measurement invariance. Although both approaches share a number of similarities, they differ in many ways [10]. For instance, standard CFA models account for the covariance between test items, whereas IRT models account for examinee item responses [11]. The main difference between these methods, however, is that the relationship between the latent construct and the true score at the item level is linear in the standard CFA framework but nonlinear in the IRT framework [10]. Indeed, standard CFA often uses linear regression, but IRT typically uses a logistic model to estimate the probability of various types of item responses and thus, to describe item functioning along a continuum [12]. Under IRT, the primary purpose of administering a psychometric test is to locate the person taking it on the latent trait scale. If such a latent trait measure can be obtained for each person taking the test, two goals can be achieved. First, the respondent can be evaluated for the severity of the characteristic of interest and second, respondents can be compared to assign severity grades [13] under the appropriate IRT model. Within the IRT family, the logistic graded-response model (GRM) is a cumulative probability model developed by Samejima [14] and designed for Likert-type items.

However, the use of traditional IRT modeling rests on the following three fundamental assumptions: unidimensionality, local independence, and monotonicity [15]. Unidimensionality means that the test measures only one dimension. Strongly related to unidimensionality, local independence means that the item should be uncorrelated after conditioning on the latent trait [16]. Finally, monotonicity means that the probability of endorsement of item response categories increases with higher levels of the latent trait. To the best of our knowledge, no study has tested GAS against the monotonicity assumption, although previous studies have reported inconsistent results regarding dimensionality and local independence. Although most studies have found support for a unidimensional factorial structure [8,17-19], this was not the case in a large Norwegian study [20], which reported a better fit for a correlated 2-factor structure that distinguished between what they interpreted as core and peripheral criteria items. Earlier work on the French and German validation of GAS conducted on the this sample reported a good fit to a unidimensional factor structure but only after allowing for the correlation of 6 error terms, which suggests some local dependencies. Of note, however, scales are rarely strictly unidimensional. Thus, it is more a matter of whether the data are adequately unidimensional to produce relatively unbiased parameters using an IRT model despite some multidimensionality [21].

Accordingly, the first aim of this study was to explore whether it is appropriate to analyze GAS using IRT modeling. IRT provides an interesting feature to investigate the equivalence in the meaning of subgroup items; when such equivalence does not hold for item parameters, it is called differential item functioning (DIF) [22]. In addition, such items are of concern because they present a potential threat to the validity of the test. Regarding the validation of GAS referred to earlier, the withdrawal item did not seem to operate equivalently for both linguistic regions [9]. Many hypotheses were invoked, including a lack of precision for this concept when applied to game use [23] and a statistically significant difference because of the large sample size. A potential limitation of the study was that only weak (equal loadings) and not strong invariance (equal loadings and intercepts or thresholds) was tested. In IRT terminology, measurement noninvariance differentiates between the nonuniform DIF (different discrimination parameter or loading) and uniform DIF (equal factor loading but different threshold). Hence, a further aim of this study was to investigate a possible DIF effect associated with the group membership within the IRT framework.

Considering the concerns and debates related to potential internet gaming addiction [24], the American Psychiatric Association recently published, in section 3 (not yet accepted conditions requiring further research) of the *Diagnostic and Statistical Manual for Mental Disorders, 5th Edition* (DSM-5) [25], the diagnostic criteria for internet gaming disorder (IGD). IGD is defined as a “persistent and recurrent use of the internet to engage in games...leading to clinically significant impairment or distress...during the past 12 months as indicated by 5 or more out of 9 criteria.” These criteria are borrowed from substance use disorder and gambling disorder criteria [26], and the adequacy of such adaptation was criticized [4,7,27-29]. In particular, high engagement in video games might not always be considered an addiction but might simply reflect elevated healthy involvement [30].

In the context of the debates related to the IGD criteria, this study aims, in addition to its primary aims, to contribute to the discussion using the data driven by the analyses on a representative sample of young adult men.

## Methods

### Participants and Procedure

The data in this study are part of a longitudinal study, the Cohort Study on Substance Use Risk Factors, designed to assess substance and game use among young Swiss men. This study protocol was approved by the Lausanne University Medical School’s Ethics Committee for Clinical Research, and we obtained written informed consent from participants. The recruitment was conducted in 3 of 6 national army recruitment centers covering 21 of 26 cantons in the French- and German-speaking regions in Switzerland. Considering that military service is mandatory for adult men in Switzerland, the sample could be considered representative of their gender and age group.

During the recruitment period (August 2010-November 2011), 15,074 men received a mandatory appointment with the army recruitment center. Of 87.87% (13,245/15,074) men who were informed about the study, 57.10% (7563/13,245) provided their written consent to participate. Questionnaires were thus sent to their private addresses to ensure complete confidentiality of participants. Overall, 79.20% (5990/7563) participants completed the assessments (3320 French-speaking and 2670 German-speaking).

### Instrument: Game Addiction Scale

We assessed participants with the 7-item version of GAS [8] translated into French and German. Because playing video games is often associated with other internet gaming-related behaviors (eg, gaming-related forums or chats and game broadcasts on apps such as YouTube) and considering that this was a large sample with diverse internet use habits who played a variety of games, the original 7-item GAS was modified to include the assessment of internet and gaming behaviors. For instance, the item “Do you play games to forget about real life?” was modified to “Do you play games or spend time on the internet to forget about real life?” Each of the 7 items was preceded by the statement “During the last 6 months, how often...” and was scored on a 5-point Likert scale (1=never, 2=rarely, 3=sometimes, 4=often, and 5=very often).

GAS was developed before the publication of DSM-5 based on a model that maintains that all addictions consist of some components (eg, salience, mood modification, tolerance, withdrawal, conflict, and relapse) [31]. The scale, nonetheless, partially covers the DSM-5 IGD criteria [32] (Table 1) [8]. However, one of the DSM-5 criteria, “jeopardized or lost a relationship, job or educational or career opportunity,” is not explicitly proposed by GAS. In addition, the GAS item “problems” related to the DSM-5 criterion of “continue despite problems” is, instead, worded in relation to a loss of interest as “Have you neglected important activities...?” (Table 1). Furthermore, the time frame used in this study was the past 6 months rather than the 1-year time frame proposed by DSM-5.

### Statistical Analysis

In this study, we used GRM because it is suitable for ordered polytomous variables [14]. GAS is a polytomous-ordered categorical scale containing 7 survey questions that measure gaming addiction on the internet. The items are labeled as salience, tolerance, mood modification, relapse, withdrawal, conflict*,* and problems and are ranked on a 5-point Likert scale from 1 (*never*) to 5 (*very often*). In GRM, the following two types of parameters were estimated: the discrimination parameter and the difficulty parameter. Because GRM is an ordered logistic model, difficulty parameters of each item were naturally estimated in the increasing order. Furthermore, the probability of observing outcome *k* or higher for item *i* and person *j* is as follows:

Pr(Y
_ij_≥k∣θ
_j_)=exp[α
_i_(θ
_j_-β
_ik_)]/{1+exp[α
_i_(θ
_j_-β
_ik_)]} with θj~N(0,1)

where α_i_ represents the discrimination of item *i*, β_ik_ is the *k* th cutoff point for item *i*, and θ_j_ is the latent trait of person *j*.

Each item varies in difficulty and shares the same discrimination parameter. Of note, the discrimination parameter (also called slope) is a measure of the differential capability of an item. A high discrimination parameter suggests that an item has a high ability to differentiate subjects. In practice, a high discrimination parameter value means that the probability of endorsing an item response increases more rapidly as the latent trait or severity increases [33].

When discrimination is high (and the item response function is steep), the item provides more information on the latent trait and the information is concentrated around item difficulty. Items with low discrimination parameters, however, are less informative, and the information is scattered along a greater part of the latent trait range. With a logistic model for the item characteristic curve (ICC), Baker [13] proposed the following different ranges of values to better interpret the discrimination parameter: 0=nondiscriminative power; 0.01-0.34=very low; 0.35-0.64=low; 0.65-1.34=moderate; 1.35-1.69=high; >1.70=very high; and + infinity=perfect.

In GRM, 2 types of parameters are estimated, the discrimination and the threshold parameters. The number of thresholds is equal to the outcome categories minus 1. In this study, we had 5 alternative responses yielding 4 thresholds. The item threshold in the GRM model refers to the level of the latent variable an individual needs to endorse the item with 50% probability [34]. In addition, we presented ICCs, which are graphical functions that represent the respondents’ latent trait as a function of the probability of endorsing the item [35]. Subsequently, ICCs were transformed into item information curves (IICs), which are a mathematical way to compute how much information each ICC can provide. Finally, IICs were summed, in turn, to obtain the test information function (TIF), which informs how well the instrument can estimate person locations. Globally, the information plots indicate the amount of psychometric information at each point along a latent severity dimension [36].

### Model Fit Analysis

**Figure 1:**
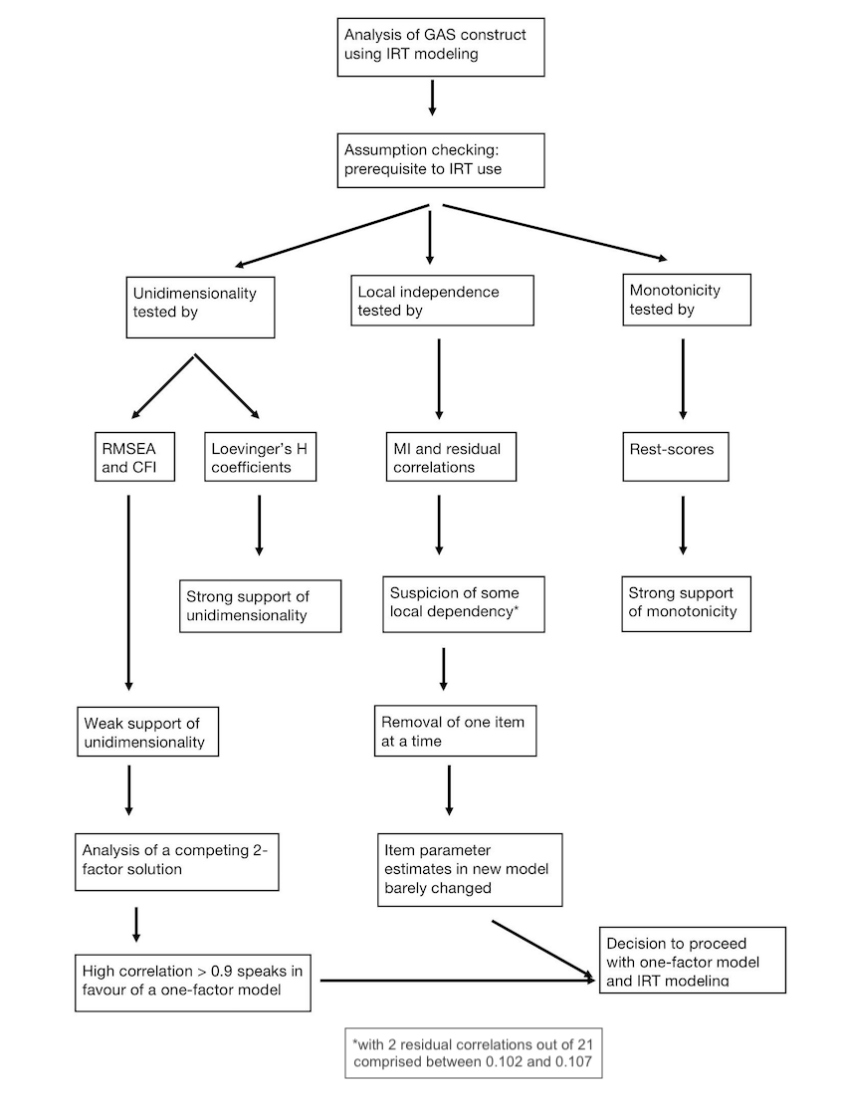
Flowchart of the steps leading to the decision to use item response theory (IRT) modeling. CFI: comparative fit index GAS: game addiction scale; MI: modification indices; RMSEA: root mean square error of approximation.

Prior to fitting a traditional item response model, a few prerequisites must be checked for the assessment of model fit, notably the assumptions of unidimensionality, local independence, and monotonicity. The flowchart in Figure 1 shows the steps leading to the use of IRT modeling.

**Table 1:** Game Addiction Scale (GAS).

How often in the last 6 months...		Answer options^a^, %					GAS items		DSM-5^b^ criteria	
		1	2	3	4	5				
**Have you thought all day long about playing a game or spending time on the internet?**								Salience		Preoccupation
	All samples	48.5	24.8	15.0	7.2	4.4				
	French	45.5	23.5	16.4	8.8	5.8				
	German	52.3	26.4	13.2	5.3	2.7				
**Have you played or stayed on the internet longer than intended?**								Tolerance		Tolerance
	All samples	36.0	21.4	24.9	12.9	4.8				
	French	31.9	20.8	27.1	14.0	6.3				
	German	41.0	22.3	22.3	11.5	2.9				
**Have you played games or spent time on the internet to forget about real life?**								Mood modification		Escape
	All samples	61.1	19.3	12.4	4.8	2.5				
	French	62.7	17.9	11.9	4.6	2.9				
	German	59.1	20.9	13.0	5.0	2.0				
**Have others unsuccessfully tried to reduce your time spent on games or the internet?**								Relapse		Unsuccessful attempts to stop or reduce
	All samples	69.8	15.7	9.9	3.4	1.3				
	French	68.5	15.8	10.5	3.6	1.7				
	German	71.3	15.6	9.1	3.1	0.8				
**Have you felt upset when you were unable to play or to spend time on the internet?**								Withdrawal		Withdrawal
	All samples	78.5	13.6	5.8	1.6	0.6				
	French	79.4	12.9	5.5	1.6	0.6				
	German	77.4	14.4	6.1	1.5	0.6				
**Have you had arguments with others (eg, family and friends) over your time spent on games on the internet?**								Conflict		Deceiving Others
	All samples	75.6	14.3	7.4	1.9	0.9				
	French	76.1	13.5	7.1	2.2	1.0				
	German	75.1	15.2	7.7	1.4	0.6				
**Have you neglected important activities (eg, school, work, and sports) to play games or spent time on the internet?**								Problems		Loss of interests
	All samples	70.0	17.0	9.2	2.6	1.3				
	French	68.1	17.2	10.2	3.0	1.4				
	German	72.2	16.7	8.0	2.1	1.0				

^a^1=never, 2=rarely, 3=sometimes, 4=often, 5=very often.

^b^DSM-5: Diagnostic and Statistical Manual of Mental Disorders, 5th Edition.

### Unidimensionality

The unidimensionality assumption suggests that the correlation among these items could be explained by a single latent factor; this assumption was assessed using 2 different approaches, the fit of a unidimensional model in the categorical confirmatory factor analysis (CCFA; declaring the data as ordinal using the weighted least square mean and variance-adjusted estimator and the Mokken scaling method, a nonparametric IRT model following an adaptation of Loevinger’s *H* coefficients [37]. When testing the unidimensional assumption for an IRT model, it is more appropriate to use CCFA than standard CFA because the former (similar to an IRT model) treats the data as categorical. In addition, the acceptable and good fit is indicated by the root mean square error of approximation (RMSEA) of <0.08 and <0.06, respectively, and the comparative fit index (CFI) values of >0.90 and >0.95, respectively [38,39]. Furthermore, the *H* coefficients express the degree of homogeneity of a set of items. When 0.3≤*H*<0.4, the scale is considered weakly unidimensional; when 0.4≤*H*<0.5, it is considered moderately unidimensional; and when *H*>0.5 [40,41], the scale is considered strong.

#### Local Independence

In local independence, it is assumed that a person’s responses to questions are not statistically related to each other when the latent trait is held constant [42], that is, the response to one item should not influence the response to another item. Moreover, because local independence is closely related to the unidimensionality assumption, some authors argued that when the latter is true, local independence is obtained [22,43]. However, we tested for local independence by evaluating the matrix of residual correlations resulting from the CCFA model. Notably, residual correlations that are >0.1 are indicative of a possible local dependence [44,45].

#### Monotonicity

The monotonicity assumption is met when the probability of endorsing a response to a test item is nondecreasing with an increase in the value of the latent construct [46]; this assumption was examined through the results of the check monotonicity function of the Mokken package. The minimum violation default value was set to 0.3, and violations greater than this value were reported. In addition, the rest-score graphs, computed as the raw scale score minus the item score for each item, also served to detect monotonicity violation patterns. Graphically, rest-scores are on the x-axis, and the proportion of respondents in each rest-score group endorsing the item is on the y-axis [47]. We used the Mokken package to plot these graphs in this study.

After we found out that the IRT assumptions were tenable, we proceeded with the estimation of the item parameters for the whole sample and the detection of a possible DIF effect by regressing the group membership on all test items and the latent symptom severity dimension.

#### Differential Item Functioning

In DIF analyses, we compared a model, in which the alpha and beta parameters were constrained to be equal for the relevant subgroups, with a model, in which the parameters were left to be free. In addition, DIF was evaluated across linguistic groups with the help of the Lordif package [48], which uses a hybrid iterative technique in an ordinal regression. Of note, this approach tests the null hypothesis that α_i_ is equal for the 2 linguistic regions (absence of the nonuniform DIF) and the null hypothesis that β_ij_ is equal (absence of the uniform DIF). Because the chi-square test is highly sensitive to sample size [49], we decided that the change in pseudo *R*^2^ also had to be a minimum of 0.035 to be flagged as a nonnegligible DIF effect [50].

#### Missing Values

The data from which this study was drawn were already analyzed for missingness in a previous study that performed hot decking [9]; this imputation technique implies that for each case with missing data, another case similar in characteristics to the case with the missing value is found but has responses for the item in question.

#### Sample Size Considerations

Sample size plays an important role in providing unbiased parameter estimates and accurate model fit information. Previous research has established guidelines concerning sample sizes needed to accurately estimate item parameters for the unidimensional GRM through simulation studies. For instance, it was reported [51] that a sample size of 375 respondents for a 15-item scale provided adequate discrimination and boundary parameter estimates. Reeve and Fayers [12] reported that GRM could be estimated with 250 respondents. However, around 500 respondents are recommended for accurate parameter estimates [12]. Stemming from a large-scale survey data, our sample widely fulfills this requirement.

In addition, we obtained all analyses and plots using the free R program (R: A language and environment for statistical computing. R Foundation for Statistical Computing, Vienna, Austria) [52]. More specifically, the Mokken package served to test the monotonicity and unidimensionality of the scale. For the detection of local dependence problems, we fit the CCFA model using the Lavaan package. In addition, we were able to estimate the IRT-GRM parameters using the latent trait models (LTM) package, and the Lordif package served to evaluate the DIF effects if any.

## Results

### Sample Characteristics

The demographic and clinical characteristics of the sample (N=5983) have been described elsewhere [9]. Table 1 presents the item response distribution by region and for the entire sample in this study.

### Unidimensionality

In a previous study [12], Velicer’s minimum average partial test and parallel analyses [53] supported the 1-factor solution; this solution was also tested by the use of a standard CFA in an asymptotic distribution-free analysis to accommodate nonnormal variables. The 1-factor solution was only supported, however, after allowing for the correlation between 6 pairs of variables, indicating a certain degree of multidimensionality. This study found similar conflicting findings. For instance, the magnitude of Loevinger’s coefficients (*H*>0.5) indicated a strong common dimension, whereas the results of a unidimensional CCFA model showed an inadequate model fit with an RMSEA value of 0.107 and a CFI value of 0.97. In addition, a competing 2-factor CCFA model, which distinguishes between core and peripheral criteria items [20], obtained a more acceptable fit (χ^2^_13_=426.0, *P*<.001; RMSEA=0.073 and CFI=0.99) but was problematic because of the correlation (>0.9) between the 2 factors being very high and >0.85 cutoff set for the discriminative validity [54]. Therefore, we proceeded with the 1-factor solution, assuming that the effects of multidimensionality were negligible.

### Local Independence

We examined local independence using modification indices and residual correlations in the CCFA model. On the one hand, the highest modification index was observed between salience and tolerance. On the other hand, having examined the concept of local independence through the residual correlation matrix, we observed that the residual correlation between salience and tolerance was 0.102, thereby marginally exceeding the cutoff value of 0.10 set by Kline. Another residual, the highest one, which also exceeded the cutoff value of 0.10, was observed between salience and conflict (0.107). These findings suggested that these item pairs (salience and tolerance as well as salience and conflict) might not be totally free of some local dependence bias. In addition, to explore the potential impact of these local dependencies, we examined whether the removal of 1 or 2 of the locally dependent items (eg, salience, tolerance, and conflict) had any noticeable effect on the size of the remaining IRT discriminative parameters in the original unidimensional model [55]. Consequently, the sizes of the remaining discriminative parameters were rather robust to such removals. The largest changes were for salience (−15%, [1.63−1.92]∕1.92) and tolerance (−14%, [1.75−2.03)∕2.03] with the removal of the tolerance and salience items, respectively. Furthermore, these modest changes supported GAS as being adequately unidimensional to obtain reasonably unbiased parameters when using traditional IRT models, despite some local dependencies.

### Monotonicity

We found no violation of monotonicity in this study because rest-score graphs (from the Mokken scale) indicated that the probability of endorsing higher categories increased along the latent trait for all items. All in all, we decided that it is acceptable to use an IRT unidimensional model on GAS.

### Item Response Theory Parameter Estimates

**Figure 2:**
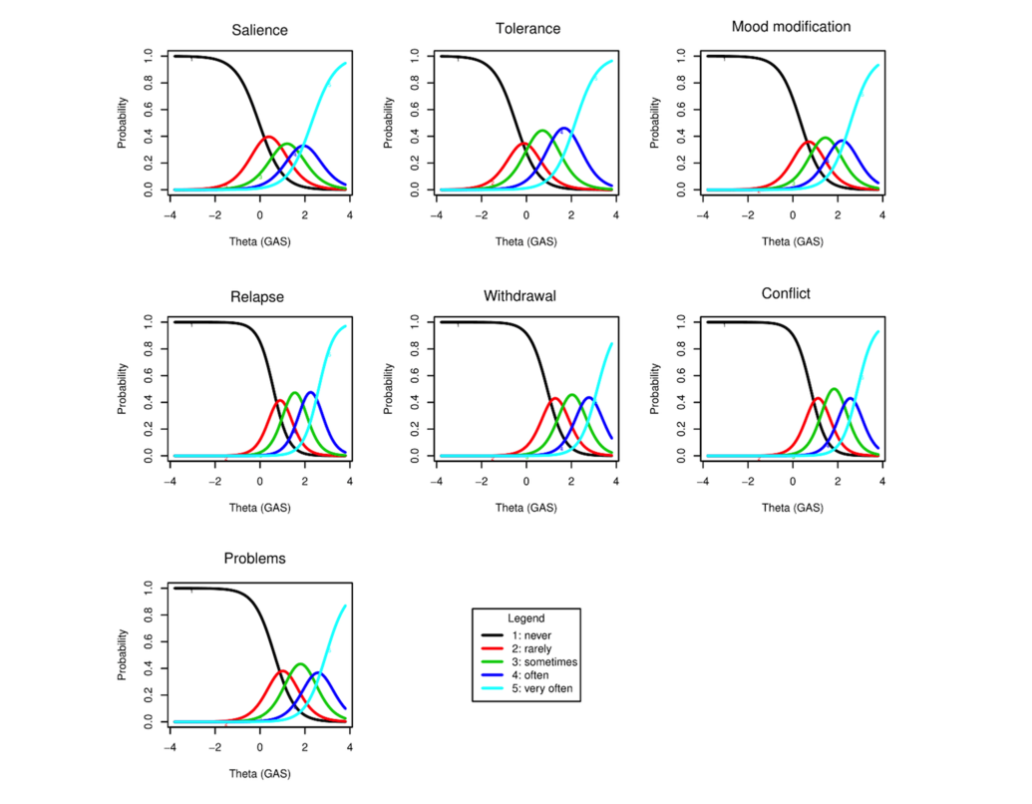
Item characteristic curves: salience; tolerance; mood modification; relapse; withdrawal; conflict; and problems. GAS: Game Addiction Scale.

**Figure 3:**
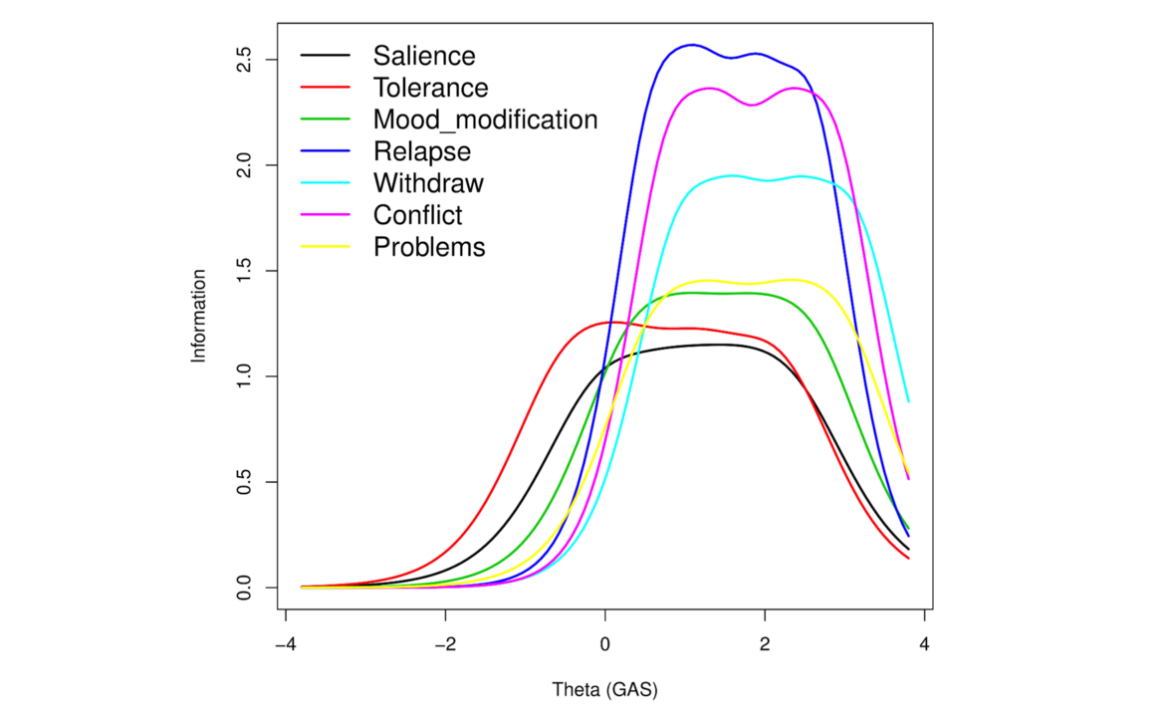
Item information curves. GAS: Game Addiction Scale.

**Figure 4:**
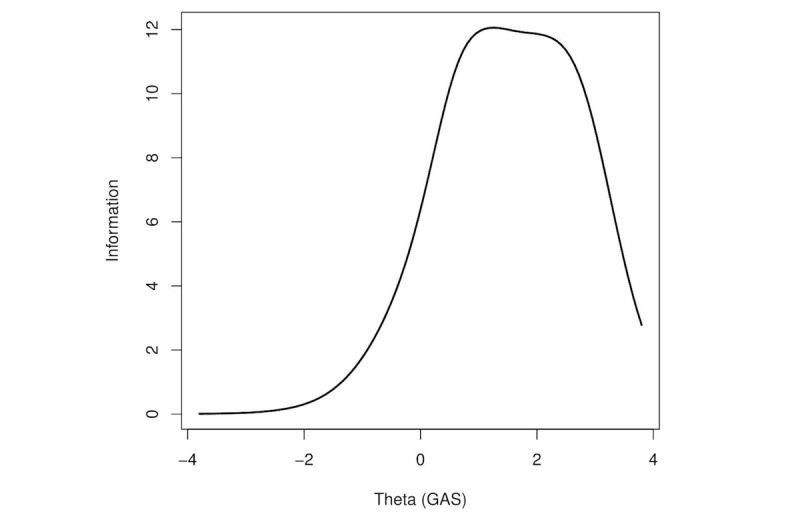
Test (scale) information function. GAS: Game Addiction Scale.

Table 2 presents results for item response modeling for GAS as well as the estimates of the parameters in the GRM. Figures 2-4 present the ICC, IIC, and TIF curves. Regarding the ranges proposed by Baker [13], we observed that all items had a very high discriminative power with a range of 1.92-2.93. In increasing order of strength, we found salience followed by tolerance, mood modification, problems, withdrawal, conflict, and relapse. Besides providing a reasonably good differentiation among individuals, large values of the parameter estimates also indicated that all items were highly related to the latent variable, gaming addiction.

In Table 2, it can also be observed that all thresholds were positive, except for those of salience and tolerance, the first threshold of which was negative. Moreover, these 2 items had the largest spread. Hence, their information functions exhibited a broader coverage on the continuum (below and above the mean), whereas the other items were better at discriminating people above the mean. In addition, we observed that all threshold parameters were not tightly clustered together, indicating that the item has adequate response options. Overall, the scale appears to cover a wide range of the item difficulty spectrum from −0.47 (with tolerance) to 3.15 (with withdrawal).

Figure 2 presents ICCs for the 7 items; these curves represent the probability that an individual selects a particular category at a given level of the latent construct. The x-axis represents the latent construct (or gaming addiction in this particular case), in which higher scores are indicative of higher game addiction. In contrast, the y-axis shows the probability of selecting each response option. In addition, each curve corresponds to one of the following 5 possible response alternatives: never, rarely, sometimes, often, and very often. Moving from left to right on the x-axis, the gaming addiction increases. Furthermore, Figure 2 shows that the response options for the respective items are monotonically related to game addiction and that each response option is most likely to be selected at some range of theta.

Consider, for example, ICCs with the largest and the smallest spread, that is, tolerance and relapse, respectively. For tolerance, subjects up to approximately 0.2 SD below the mean were more likely to endorse response category 1 (never); from 0.2 SD below the mean to 0.1 SD above, they were more likely to endorse category 2 (rarely); and from 0.1 to 1.2 SD above the mean, they were more likely to respond to category 3 (sometimes). In addition, from 1.2 to 2.0 SD above the mean, they exhibited the highest likelihood of endorsing category 4 (often). Finally, subjects most likely to choose category 5 (very often) were those with the intensity of gaming disorder symptoms of >2.0.

As GRM is defined in terms of cumulative probabilities, we also performed cumulative comparisons. The difficulties represented a point at which a person with *θ=b*_ik_ had a 50% chance of responding in category *k* or higher [56]. For example, looking at the estimated parameters for tolerance, we observed that a person with *θ*=−0.47 has a 50% chance of answering 1 versus ≥2 and a person with *θ*=0.24 has a 50% chance of answering 1 or 2 versus ≥3. Similarly, a person with *θ*=1.18 has a 50% chance of answering 1, 2, or 3 versus ≥4, and a person with *θ*=2.17 has a 50% chance of answering 1, 2, 3, or 4 versus 5. We noted that the ratings for tolerance span a broad range of the latent trait and that its discrimination parameter was high.

For relapse, subjects up to 0.8 SD above the mean were more likely to endorse response category 1 (never); subjects from 0.8 to 1.2 SD above the mean were more likely to endorse category 2 (rarely); and subjects from 1.2 to 1.8 SD above the mean were more likely to respond to category 3 (sometimes). From 1.8 to 2.5 SD above the mean, they exhibited the highest likelihood of endorsing category 4 (often); from 2.5 SD above the mean, they were more likely to choose category 5 (very often). With the highest discrimination parameter, we noted that the curves for relapse were more peaked than for tolerance and more concentrated toward the upper end of the trait.

**Table 2:** Estimates of discrimination and severity parameters for the Game Addiction Scale under the graded-response model with the LTM package.

Item	Discrimination, α_i_^a^	Severity				
		β_i1_	β_i2_	β_i3_	β_i4_	Spread
Salience	1.92	−0.04	0.83	1.58	2.29	2.33
Tolerance	2.03	−0.47	0.24	1.18	2.17	2.64
Mood modification	2.13	0.35	1.06	1.83	2.56	2.21
Relapse	2.93	0.59	1.20	1.90	2.61	2.02
Withdrawal	2.56	0.92	1.64	2.42	3.15	2.23
Conflict	2.83	0.79	1.45	2.23	2.88	2.09
Problems	2.19	0.65	1.38	2.23	2.93	2.28

^a^α_i_ reflects the ability of item i to discriminate between different levels of game addiction severity (θ).

^b^β_ik_ is the *k* th cutoff point for item *i*. It is interpreted as the standardized level of game addiction severity where subsequent response options become more probable than the previous option.

Figure 3 plots IICs of the 7 items. The shape of an IIC was determined both by its discrimination and threshold parameters; however, the steepness of the curves was determined by the magnitude of the discrimination index. Salience, tolerance, mood modification, and problems were less steep than relapse, conflict, and withdrawal, but they covered a wider range of the item severity spectrum. In turn, the latter best discriminated the population for the latent trait at a higher level.

Figure 4 presents TIF, which is the condensed information of each item in Figure 3. Applying the formula [12] reliability=1 − (1∕information), we observed that the scale reliably assessed a wide range of individuals below and above the average. For instance, information scores of 5-12, which translate to a reliability range of 0.80-0.92, corresponded to participants from 0.3 SD below to 2.5 SD above the mean.

**Figure 5:**
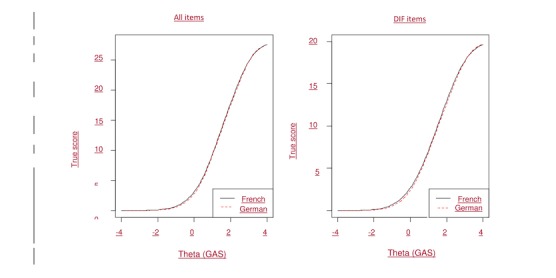
Test characteristic curve: all items (left) and differential item functioning (DIF) items (right). GAS: Game Addiction Scale.

### Differential Item Functioning Parameter Estimates

The results obtained with Lordif software showed that 5 of 7 items (ie, salience, tolerance, mood modification, withdrawal, and conflict) were flagged for DIF using the change in the chi-square. Of note, relapse and problems were not flagged as DIF and thus, used as anchors. The nonuniform DIF, that is, a different slope or discrimination parameter between the 2 linguistic regions, was present in salience, withdrawal, and conflict, whereas the uniform DIF, resulting in different severity parameters, was observed in tolerance and mood modification. After the inspection of the pseudo *R*^2^ (not shown here), all could be regarded as negligible when using Jodoin and Gierl’s criteria (the largest being 0.0073) [50]. In addition, the test characteristic curves for all items (Figure 5, on left) and the DIF items only (Figure 5, on right) revealed that the impact of the DIF items was trivial at the scale level because the expected total score was virtually identical for the 2 linguistic groups along the whole latent trait continuum.

## Discussion

### Item Response Theory Modeling

In this study, using IRT modeling, we investigated the psychometric properties of GAS for the amount of information provided by the 7 items and the severity of the latent trait being measured. Although the monotonicity assumption was satisfied, the fit of the unidimensional model was somewhat unsatisfactory owing to the fact that 3 items appeared to be locally dependent. Although these dependencies had some impact on the IRT discriminative parameters, their impact on the performance of new coefficients was not large (maximum 15%). Indeed, referring to Baker’s cutoff points, their estimates, except one, remained in the “very high range” category. In addition, when we modeled a 2-factor solution, the high correlation between the 2 dimensions was a matter of concern and ignoring this finding would have undermined their discriminative validity. Satisfied by the strong Loevinger’s *H* coefficients, suggesting the occurrence of a strong primary factor, we decided to retain the 1-dimension model and concluded that it is reasonable to analyze GAS with a traditional IRT model. However, it is important to emphasize that the practical impact of ignoring multidimensionality probably depends on the intended use of the scale. Although the local dependencies shown in this study will probably exert a negligible impact on the scaling of individuals, the available research suggests that even minor violations of unidimensionality can exert an important impact on various aspects such as score reliability, differential functioning, and linking [57-59].

All items had high discrimination parameters and as a set, these items differentiated across a reasonable range of the trait. In accordance with Baker’s interpretation, their discriminative power was very high [13] with the estimated parameters ranging from 1.92 to 2.93. Overall, the severity parameters (β_1_-β_4_), which reflect the range of the underlying construct, were between −0.47 and 3.15 for the whole sample, implying that the items show reasonable variability for the endorsement of response categories. Furthermore, no *null categories* existed because all item response categories were chosen by the respondents, null categories being referred to as “never chosen categories.” Reportedly, none of the items in response categories seem to be superfluous owing to the fact that their response occupied a distinct portion of the ability continuum [60].

### Internet Gaming Disorder Criteria Debate

Theoretical debate is ongoing about the IGD criteria in consideration of their ability to capture the features of addictive internet gaming and their potential tendency to conflate passion (ie, healthy repeated use) and disorder (ie, pathological addictive use). The following 4 criteria, described by some authors of the core addiction criteria [20,30], received more consensus than the other criteria: unsuccessful attempts to reduce or stop [4]; loss of interest in previous hobbies or activities [32,61,62]; continuation despite problems [4,30,62]; and jeopardized or lost a relationship, job, or educational or career opportunity [62]. When observing such criteria, careful attention must be paid to possible coping motives (ie, related to a depressive disorder) before attributing any such symptoms to addictive behavior [61,63].

The following 5 criteria are more controversial:

1. Preoccupation (being absorbed by gaming and thinking about it): this criterion, thought to be related to cognitive salience, is considered a core criterion by some authors [30,64] but not others [20,61,65]. Preoccupation is commonly reported among high achievers [26,66] and is supposed to be common for gamers because of the social features of the games and flow-related engagement [65,67].
2. Withdrawal: Considered to be a core symptom in some studies [20,30], this criterion has, nonetheless, come under criticism (ie, difficulty distinguishing it from irritability related to the involuntary discontinuation of gaming). The withdrawal symptoms described for IGD were mostly irritability, restlessness, and sadness [68].
3. Tolerance: This criterion refers to the need to increasingly engage in games to feel as though one has played enough. Progression is, however, a part of the game process. This criterion is, therefore, difficult to conceptualize for IGD [69].
4. Escape: Despite the association between game involvement and escape motives [2,70,71], the specificity of this criterion and its link with possible primary disorders (ie, depression) has been discussed [4,26,64,66,72]. In some, but not all [65], IGD-related studies, low diagnostic accuracy was observed for this criterion [61,64].
5. Deceiving others (such as lying to relatives related to the number of games): This criterion is related to “excessive gaming despite problems” and conflicts. Considered as core by some authors [20,30], deceiving others is, however, sensitive to cultural aspects and interactions with relatives and age probably lead to low accuracy of the criterion in some adult studies [66].

Most debates related to the validity of the criteria were theoretically based and insufficiently data-driven [4,73] and thus, more empirical work is warranted [74]. Kiraly et al [62] examined how each IGD criterion performs at different severity levels using an IRT approach and demonstrated that some criteria, such as preoccupation, escape, continue despite problems, and jeopardized or lost a relationship, were endorsed more frequently in less severe IGD stages, whereas other criteria, such as tolerance, unsuccessful attempts to stop or reduce, loss of interest in previous hobbies or activities, and deceiving others, were reported only in more severe cases. However, the study was exposed to self-selected bias because of the Web-based recruitment of a convenience sample [75].

### Reappraisal of Internet Gaming Disorder Criteria Using Game Addiction Scale as Proxy

Figure 4 shows that the information provided by GAS is reliable about respondents who are located between 0.3 SD below and 2.5 SD above the mean, suggesting that the scale does a good job of differentiating individuals below and above the average even though it is more precise at a higher level above the mean. Specifically, relapse (unsuccessful attempts to stop or reduce), conflict (deceiving others), withdrawal and problems (loss of interests) were the GAS items with a higher ability to discriminate IGD (endorsed more frequently in more severe IGD stages), whereas the items related to tolerance, salience (preoccupation), and mood modification (escape) were endorsed more widely among participants (included in less severe IGD stages). The results regarding preoccupation and escape were in concordance with those reported in previous studies [62,64], which showed large endorsement of the criteria. As reported in other studies, loss of interests [61,62], unsuccessful attempts to reduce or stop [62], deceiving others [62], and withdrawal [61,62] were more endorsed among participants with more severe IGD.

In contrast to the findings of this study, tolerance was endorsed by more severe cases in other studies [61]. This contradiction could be attributed to the differences in samples or in the wording of the criteria across scales (eg, “Have you ever felt the need to play more often or played for longer periods to feel that you have played enough?” vs “Have you played longer than intended?” in GAS). The wording used in GAS could realistically be interpreted as a form of loss of control or a form of enthusiasm related to the flow [76] induced by the mechanisms of game progression [69]. The wording used for this item in GAS is, perhaps, not entirely successful in capturing the intended meaning of tolerance [32], which might also be part of the reason that we found some local dependence between salience and tolerance in this study.

This study highlights that the IGD condition, as assessed by GAS and the proposed IGD criteria, involves different symptoms, some of which were widely disseminated across the sample and others that were characteristic of disorder severity. However, the GAS items differ from the IGD criteria in several ways. Hence, GAS has to be considered as a proxy measure of the IGD criteria and the findings must be interpreted accordingly.

As found in other studies, the preoccupation [61,62,65] and escape [64,66] criteria exhibited lower discriminatory power than that exhibited by other items. The deceiving other items had good discriminant capacity in this study and others [62], whereas some studies reported low diagnostic accuracy for this criterion [66]. This study was conducted on young adult men, and one may hypothesize that this item is more sensitive to differences in cultural contexts, family contexts, and age groups. In addition, discrepancies between the study results for this criterion could be attributed to differences in item wording across studies (“Have you had arguments with others?” in this study). Furthermore, we cannot exclude that the discriminative ability of the item is inflated in this study because of local dependencies in the model.

### Differential Item Functioning

DIF occurs when items have a different relation with the construct in different subgroups; in our case, it is linguistic status. In this study, the discrimination and threshold parameters were very similar between the 2 linguistic groups as the uniform DIF and nonuniform DIF were found to be negligible, as shown by the weak pseudo *R*^2^. A change in beta showed no significant effect size, except for the withdrawal item, which was just above the 0.01 cutoff; this is the same item that was flagged for measurement invariance in a previous validation of this scale with the AMOS software. However, as can be seen in Figure 5, the curves are superposed. As we expected, the conclusions drawn from standard CFA analyses concerning the measurement invariance between the 2 linguistic regions are unambiguously supported by IRT analyses.

### Limitations

This study has several limitations. First, although the sample is representative, it included only young men of about the same age group (almost 99% of them were between 18 and 24 years of age) from Switzerland, thereby limiting the generalizability of the results. Even though the military service is not mandatory for women in Switzerland, it enrolls a marginal number of them each year on a voluntary basis. Because of this marginal number and, more importantly, because no official figures of the female representation were available during the recruitment period, female army recruits were not considered in this study, further limiting the generalizability. However, the sample recruitment allowed us to overcome the self-selection biases reported in other studies [62]. Second, another limitation is related to the use of self-reported questionnaires with possible differences in understanding of questions, desirability bias, and recall bias and the difficulty in assessing the context of a given behavior. Other limitations of the study are directly related to the GAS instrument. In this study, several DSM-5 criteria, such as the loss of opportunities and relationships, were not included in GAS nor were other possibly important criteria for assessing IGD, such as craving or immersion. In addition, the time frame differed (6 months) than that proposed by DSM-5 (12 months). Furthermore, the study did not directly assess the internet-based or game activities used by participants. Thus, for example, we were not able to differentiate one game activity from another or a specific game activity from other types of internet use behavior, although the participants’ answers might have related to a specific activity or a combination of activities. However, the advantage of such an approach is that other internet gaming-related activities, which can be time-consuming and performed in excess (eg, game broadcasts), are covered by the items.

Despite the variability across game mechanisms [77], it appears that video games are addictive among some users through refined rewards and processes contributing to the loss of control over game use [78]. In consideration of such similarities between the behavior associated with video games and that associated with other games, numerous studies have assessed games in general without focusing on a specific gaming behavior [62,79]. In addition, previous studies showed the suitability of assessing different internet behaviors (ie, internet gambling and internet gaming) using similar scales [80], whereas other studies concluded the differences between the problematic internet use and online gaming using different assessment tools and finding mostly between-group gender differences [81]. Hence, further studies with IRT analyses are warranted to increase our understanding of the similarities and differences across different types of excessive internet and game use behaviors.

### Conclusions

This study partly supports the relevance of using IRT to further establish the psychometric properties of the GAS items. With respect to an overall picture of the symptoms assessed by GAS, relapse, conflict, withdrawal, and problems were endorsed more frequently in more severe IGD stages, whereas the items related to tolerance, preoccupation, and mood modification were endorsed more widely, including among participants in less severe IGD stages. However, these findings must be considered with caution because GAS measures something akin to the IGD criteria but does not measure these criteria *per se*.

## Abbreviations

CCFA: categorical confirmatory factor analysis
CFA: confirmatory factor analysis
CFI: comparative fit index
DIF: differential item functioning
DSM-5: Diagnostic and Statistical Manual for Mental Disorders, 5th Edition
GAS: Game Addiction Scale
GRM: graded-response model
ICC: item characteristic curve
IIC: item information curve
IGD: internet gaming disorder
IRT: item response theory
RMSEA: root mean square error of approximation
TIF: test information function
